# Peak functional ability and age at loss of ambulation in Duchenne muscular dystrophy

**DOI:** 10.1111/dmcn.15176

**Published:** 2022-02-14

**Authors:** Alberto A. Zambon, Vandana Ayyar Gupta, Deborah Ridout, Adnan Y. Manzur, Giovanni Baranello, Federica Trucco, Francesco Muntoni, The UK Northstar Clinical Network, Sandya Tirupath, Melanie Douglas, Jaci McFetridge, Deepak Parasuraman, Zoya Alhaswani, Heather McMurchie, Rosanna Rabb, Anirban Majumdar, Kayal Vijayakumar, Sam Amin, Faye Mason, Claire Frimpong‐Ansah, Frances Gibbon, Bethan Parson, Karen Naismith, Julie Burslem, Alex Baxter, Clare Eadie, Iain Horrocks, Marina Di Marco, Anne‐Marie Childs, Lindsey Pallant, Stefan Spinty, Alison Shillington, Sarah Gregson, Laura Cheshman, Elizabeth Wraige, Vasantha Gowda, Heinz Jungbluth, Jennie Sheehan, Imelda Hughes, Sinead Warner, Volker Straub, Michela Guglieri, Anna Mayhew, Gabby Chow, Sarah Williamson, Tracey Willis, Richa Kulshrestha, Nicholas Emery, Sithara Ramdas, Hayley Ramjattan, Christian de Goede, Andrea Selley, Min Ong, Kay White, Marjorie Illingworth, Michelle Geary, Jenni Palmer, Cathy White, Kate Greenfield, Gemunu Hewawitharana, Yvonne Julien, Elma Stephens, Jane Tewnion, Gautam Ambegaonkar, Deepa Krishnakumar, Jacqui Taylor, Catherine Ward, Tracey Willis, Elizabeth Wright, Claire Rylance

**Affiliations:** ^1^ Dubowitz Neuromuscular Centre UCL Great Ormond Street Institute of Child Health & Great Ormond Street Hospital London UK; ^2^ Neuromuscular Repair Unit Institute of Experimental Neurology (InSpe) Division of Neuroscience IRCCS Ospedale San Raffaele Milan Italy; ^3^ Population, Policy and Practice Research and Teaching Department UCL Great Ormond Street Institute of Child Health London UK; ^4^ NIHR Great Ormond Street Hospital Biomedical Research Centre London UK; ^5^ Children’s Sleep Medicine Evelina Children Hospital ‐ Paediatric Respiratory Department Royal Brompton Hospital Guy’s and St Thomas’ Trust London UK

## Abstract

**Aim:**

To correlate the North Star Ambulatory Assessment (NSAA) and timed rise from floor (TRF) recorded at age of expected peak with age at loss of ambulation (LOA) in Duchenne muscular dystrophy (DMD).

**Method:**

Male children with DMD enrolled in the UK North Start Network database were included according to the following criteria: follow‐up longer than 3 years, one NSAA record between 6 years and 7 years 6 months (baseline), at least one visit when older than 8 years. Data about corticosteroid treatment, LOA, genotype, NSAA, and TRF were analysed. Age at LOA among the different groups based on NSAA and TRF was determined by log‐rank tests. Cox proportional hazard models were used for multivariable analysis.

**Results:**

A total of 293 patients from 13 different centres were included. Mean (SD) age at first and last visit was 5 years 6 months (1 year 2 months) and 12 years 8 months (2 years 11 months) (median follow‐up 7 years 4 months). Higher NSAA and lower TRF at baseline were associated with older age at LOA (*p*<0.001). Patients scoring NSAA 32 to 34 had a probability of 0.61 of being ambulant when older than 13 years compared with 0.34 for those scoring 26 to 31. In multivariable analysis, NSAA, TRF, and corticosteroid daily regimen (vs intermittent) were all independently associated with outcome (*p*=0.01).

**Interpretation:**

Higher functional abilities at peak are associated with older age at LOA in DMD. This information is important for counselling families. These baseline measures should also be considered when designing clinical trials.

## Graphical abstract

In Duchenne muscular dystrophy, we lack data linking early motor abilities to loss of ambulation (LOA). In this study, we investigated the relationship between two functional scores (i.e. North Star Ambulatory Assessment [NSAA] and Timed Rise from Floor [TRF]) recorded at the time of expected peak and LOA. Both higher NSAA scores and lower TRF were associated with later age at LOA. This information is important for counselling families and should also be considered when designing clinical trials.

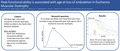

**Abbreviations**
DMD: Duchenne muscular dystrophy
LOA: Loss of ambulation
NSAA: North Star Ambulatory Assessment
TRF: Timed rise from floor

**What this paper adds**

- Functional measures recorded between 6 and 7 years 6 months are associated with long‐term outcome.
- North Star Ambulatory assessment and timed rise from floor were associated with age at loss of ambulation.

Duchenne muscular dystrophy (DMD) is an X‐linked recessive neuromuscular disorder affecting approximately 1 in 3500 to 5050 live male births^1^, ^2^, ^3^ and is caused by mutations in the gene coding for the dystrophin protein. DMD is characterized by progressive weakening of skeletal, respiratory, and cardiac muscles.

From early childhood, young males with DMD perform worse than typically developing peers in terms of motor abilities.^4^ These can be measured by a 34‐point functional scale specifically developed for DMD, the North Star Ambulatory Assessment (NSAA). While NSAA scores in typically developing males are expected to peak at 4 years of age, in DMD this occurs at a median age of 6 to 7 years, after which the scores plateau and subsequently decline.^5^, ^6^

Although a phenotypic variability exists, DMD is inexorably a progressive disease, leading to inevitable loss of ambulation (LOA) and wheelchair dependency by a median age of 10 years in corticosteroid‐naive patients (meaning those individuals who had never received steroid treatment), with a delay of 2 to 4 years when long‐term corticosteroid treatment is administered.^7^, ^8^, ^9^

Most research currently focuses on 12 to 36 months’ trajectory of different motor^5^, ^10^, ^11^, ^12^, ^13^, ^14^ and respiratory^15^, ^16^, ^17^ outcome measures providing comparative data for ongoing interventional trials, in which the typical duration ranges between 48 and 144 weeks.^18^ However, information about early prognostic factors of long‐term decline is lacking, for instance the relation between the highest achieved functional ability and age at LOA. This is relevant considering that the DMD spectrum encompasses clusters of male children with different trajectories of ambulatory function,^19^ and that such variability poses significant challenges when designing clinical trials. In addition, information about future prognosis would help counsel families, anticipate complications, and tailor the care for patients in a cost–benefit manner. A recent study described four latent class groups based on the age at which NSAA total score fell below the fifth centile (namely ~10, 12, 14, and >15 years).^6^ However, while patients belonging to each group presented with different characteristics at first NSAA assessment (e.g. age at corticosteroid initiation and functional scores), the study was not designed to establish which baseline factors were associated with subsequent age at LOA.

Here we aim to assess whether two commonly used functional scores (namely NSAA total score and timed rise from floor [TRF]) recorded at the age of expected best motor performance are associated with age at LOA in male children with DMD treated according to the current standards of care.

## METHOD

### Study design and selection of patients

This was a retrospective cohort study on patients affected by DMD, whose data were prospectively collected according to a predefined protocol. Through the DMD UK NorthStar Network (a national network of 23 UK neuromuscular centres) database, patients’ data meeting the following criteria were included in the analysis: (1) at least one visit when older than 8 years; (2) at least 3 years of follow‐up; (3) at least two visits recorded in the data set; (4) availability of at least one NSAA score recording between 6 years and 7 years 6 months of age. The reason why we selected criterion (4) was that previous studies showed that NSAA is expected to peak between 6 years 4 months and 7 years of age^5^, ^6^ and, more precisely, at a mean age (SD) of 6 years 10 months (8 months).^20^ Patients exposed to drugs potentially affecting LOA (e.g. ataluren, exon skipping) and patients with substantial learning difficulties unable to collaborate with the assessment were excluded (NorthStar collects prospectively substantial learning difficulties affecting the possibility of performing a reliable assessment).

### Procedure

The following variables were collected and reviewed: date of birth, height, weight, *DMD* gene mutation (thereafter genotype), corticosteroid treatment, NSAA total score and TRF, age at LOA (defined by the inability to walk 10 steps independently), age at last ambulant visit (for those still ambulant or those lacking information about age at LOA), and presence of long bone fractures near to the age at LOA. *DMD* genotypes were grouped according to amenability to specific exon skipping as those who had already achieved clinical application or were in advanced preclinical studies.

### NSAA and functional scores

The NSAA is a validated, 34‐point, functional scale, developed for ambulant male children with DMD. The scale is suitable for multicentric studies and is widely used in clinical settings and as a primary or secondary endpoint in clinical trials.^5^, ^21^, ^22^, ^23^ The NSAA includes 17 items, two of which are timed. Specifically, we analysed TRF, measured in seconds.^24^ The highest NSAA score recorded within the 6 years to 7 years 6 months window (and its concomitant TRF recording) was used as the baseline measurement.

### Calculation of corticosteroid exposure

Longitudinal corticosteroid treatment was reviewed for each patient. Corticosteroid type, regime, and dose per kilogram for each visit were recorded. We defined corticosteroid regime as ‘predominantly daily’ or ‘predominantly intermittent’ when one regimen was used for more than 50% of the time before LOA. According to DMD guidelines, the recommended daily dose pro‐kilo for prednisolone and deflazacort is 0.75mg/kg and 0.9mg/kg respectively. In clinical practice though, corticosteroid dose and regimens are often adjusted owing to side effects (e.g. weight gain, behavioural changes). This has not always been clearly depicted in previous studies, where information has often been limited to corticosteroid exposure ‘yes or no’ or corticosteroid regimen used. We therefore calculated the corticosteroid dose pro‐kilo at baseline expressed as the percentage of the optimal dose, namely 0.75mg/kg and 0.9mg/kg for prednisone and deflazacort respectively.

### Statistical analysis

Descriptive statistics, including mean, standard deviation, or median and interquartile range (IQR) were generated for all continuous measures as appropriate and categorical data were summarized as frequency and percentage. Spearman’s correlation test was used to determine correlations for skewed variables. We used log‐rank tests and Cox regression analysis to explore the relation between patient baseline characteristics and age at LOA. For those patients who had not yet lost ambulation, we used their age at last ambulant visit as a censored measure. The proportional hazard assumption was checked for all models.

We performed univariate analysis to explore the relations between the following baseline characteristics and age at LOA: NSAA score (first as actual NSAA and then categorized as <22, 22–25, 26–28, 29–31, and 32–34); TRF (first as actual TRF and then categorized as ≤3.5s, 3.6–5s, and >5s); corticosteroid regimen at baseline (intermittent vs daily); and genotype. Patients amenable to two different exon skipping were included in both groups (e.g. exon 44 and 55, exon 51 and 53, and exon 50 and 52; see Figure S1). To aid interpretation of results, we established NSAA and TRF categories based on clinical meaningfulness, namely intervals that reflected a significant functional difference. For instance, while a 1‐unit change in NSAA has little clinical meaning, a 3‐point difference reflects a significant change in function which can be easily understood by clinicians, and which meets the definition of the minimal clinically important difference. Only those measures that were significantly associated with LOA in the univariate analysis or known to be clinically meaningful were included in the multivariable analysis (namely NSAA, TRF, and corticosteroid regimen). For multivariable analysis results, we present hazard ratios and 95% confidence intervals (CIs) and use a single reference category to calculate hazard ratios. In addition, we present probability estimates for age at LOA with 95% CIs. All analyses were conducted using R (R Foundation for Statistical Computing, Vienna, Austria). Results were considered significant when *p*<0.05.

## RESULTS

### Population characteristics

A total of 293 patients from 13 different centres were included in this study. The mean age at first and last visit was 5 years 6 months (1 year 2 months) and 12 years 9 months (2 years 10 months) respectively. Two hundred and eighty‐six patients received corticosteroid treatment before losing ambulation; seven were corticosteroid‐naive. The mean (SD) age at initiation of corticosteroid treatment was 5 years 6 months (1 year). For 109 out of 286 (38%) patients, corticosteroid regime and/or corticosteroid type was subsequently changed before losing ambulation (‘corticosteroid‐switcher’). Including corticosteroid‐switchers, 160 (56%) patients were predominantly treated with daily corticosteroid regimen, and 83 (29%) intermittently. Cohort characteristics are presented in Table 1 and details about the selection of patients and genotypes in Figure S1.

**TABLE 1:** Demographics, corticosteroid treatment, and baseline functional abilities

	Patients (*n*=293)
Age at first visit, years:months	
Mean (SD)	5:6 (1:2)
Median (IQR)	5:6 (4:10–6:4)
Age at last visit, years:months	
Mean (SD)	12:8 (2:11)
Median (IQR)	12:8 (10:1–15:1)
Years:months of follow‐up	
Mean (SD)	7:4 (2:10)
Median (IQR)	6:11 (5:0–9:2)
Ambulatory status at last visit, *n* (%)	
Ambulatory	133 (45)
Non‐ambulatory	160 (55)
Age at corticosteroid initiation, years:months	
Mean (SD)	5:6 (1:0)
Median (IQR)	5:5 (4:10–6:1)
Corticosteroid regimen before LOA, *n* (%/293)	
Corticosteroid‐naive	7 (2)
Corticosteroid‐treated	
Daily	101 (34)
Intermittent	52 (18)
n/a	24 (8)
Switcher	109 (38)
Predominantly daily	59/109
Predominantly intermittent	31/109
50/50	14/109
n/a	5/109
Corticosteroid‐switcher before LOA, *n* (%/109)	109
Switch corticosteroid type	29 (28)
Switch corticosteroid regimen	58 (55)
Switch corticosteroid type and regimen	19 (19)
Stopped	3 (3)
Corticosteroid mean annual dose pro‐kilo before LOA, mean % of desired (SD)^a^	
Daily regimen	74 (14)
Intermittent regimen	41 (4)
Switcher	
Predominantly daily	71 (12)
Predominantly intermittent	51 (8)
Age at baseline, years:months	
Mean (SD)	6:10 (0:6)
Median (IQR) NSAA total score at baseline	
Corticosteroid‐naive^b^ (*n*=30)	24 (19–30)
Corticosteroid‐treated (*n*=263)	27 (22–31)
Median (IQR) time to rise at baseline, s	
Corticosteroid‐naive^b^ (*n*=30)	5.1 (4.1–7.4; range 1.8–17.3)
Corticosteroid‐treated (*n*=250)	4 (3.2–5.5; range 1.2–22)

^a^ The mean annual corticosteroid dose pro‐kilo was calculated from the time of corticosteroid initiation to the age at loss of ambulation (LOA) or to the age of 11 years if patients were still ambulant. Considering 0.75mg/kg and 0.9mg/kg 365 days a year as the optimal dose (100%), we calculated the percentage of desired annual dose per year and subsequently the average percentage of desired annual corticosteroid dose before LOA, such that if 0.75mg/kg prednisolone intermittent regimen were used this would be equal to 50% of optimal dose.

^b^ Corticosteroid‐naive at baseline. n/a, data not available.

### Baseline assessment: functional scores and corticosteroid treatment

The mean (SD) age at baseline was 6 years 10 months (6 months). The median (IQR) highest NSAA score at baseline was 27 (22–31) and the mean (SD) TRF was 4.9 seconds (2.9) (Fig. 1a). There was a significant negative correlation between total NSAA and TRF at baseline (Spearman’s *r*=−0.64; *p*<0.001) (Fig. 1b). Information about corticosteroid regimen at baseline was available for 224 out of 293 patients. Of the remaining 69 patients, 30 were corticosteroid‐naive (23 of whom were subsequently started on corticosteroids). There was no correlation between corticosteroid dose pro‐kilo and total NSAA score at baseline (Fig. 1c).

**GURE 1:**
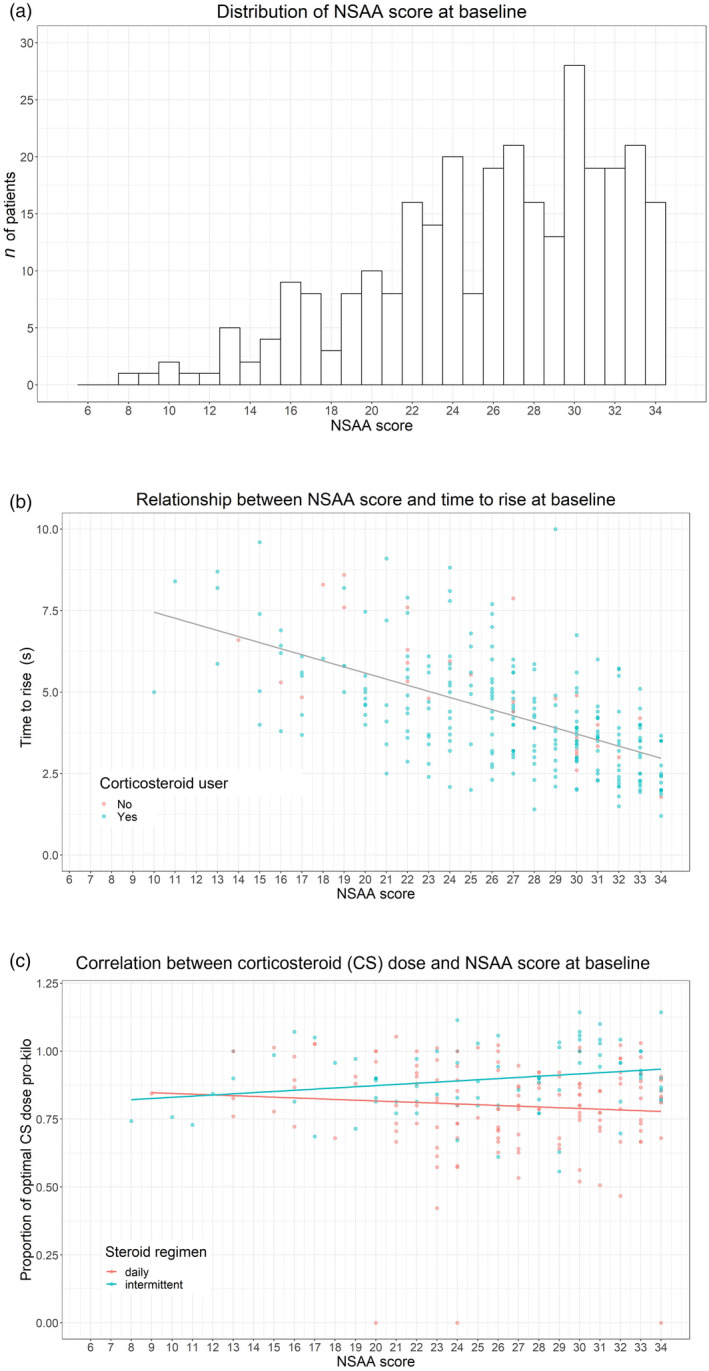
(a) Frequency of North Star Ambulatory Assessment (NSAA) scores at baseline. (b) Correlation between NSAA and timed rise from floor (TRF) at baseline. (c) Correlation between corticosteroid dose/regimen and NSAA score at baseline

Before LOA, 211 (72%) patients were on prednisolone (116 on a mean dose of 0.6mg/kg daily; 77 on a mean dose of 0.6mg/kg intermittent 10d on/off), 32 (11%) on deflazacort (25 on a median dose of 0.7mg/kg daily, six on a median dose of 0.7mg/kg intermittent), 20 on unspecified corticosteroid type, and 30 were corticosteroid‐naive.

### LOA

A total of 160 out of 293 patients lost ambulation. Twenty‐two of the 160 patients (13.75%) had a long bone fracture within 6 months before LOA. The overall estimated median age at LOA (IQR) was 11 years 8 months (10 years 1 month–14 years 5 months).

#### Relation between baseline functional abilities and LOA

**FIGURE 2:**
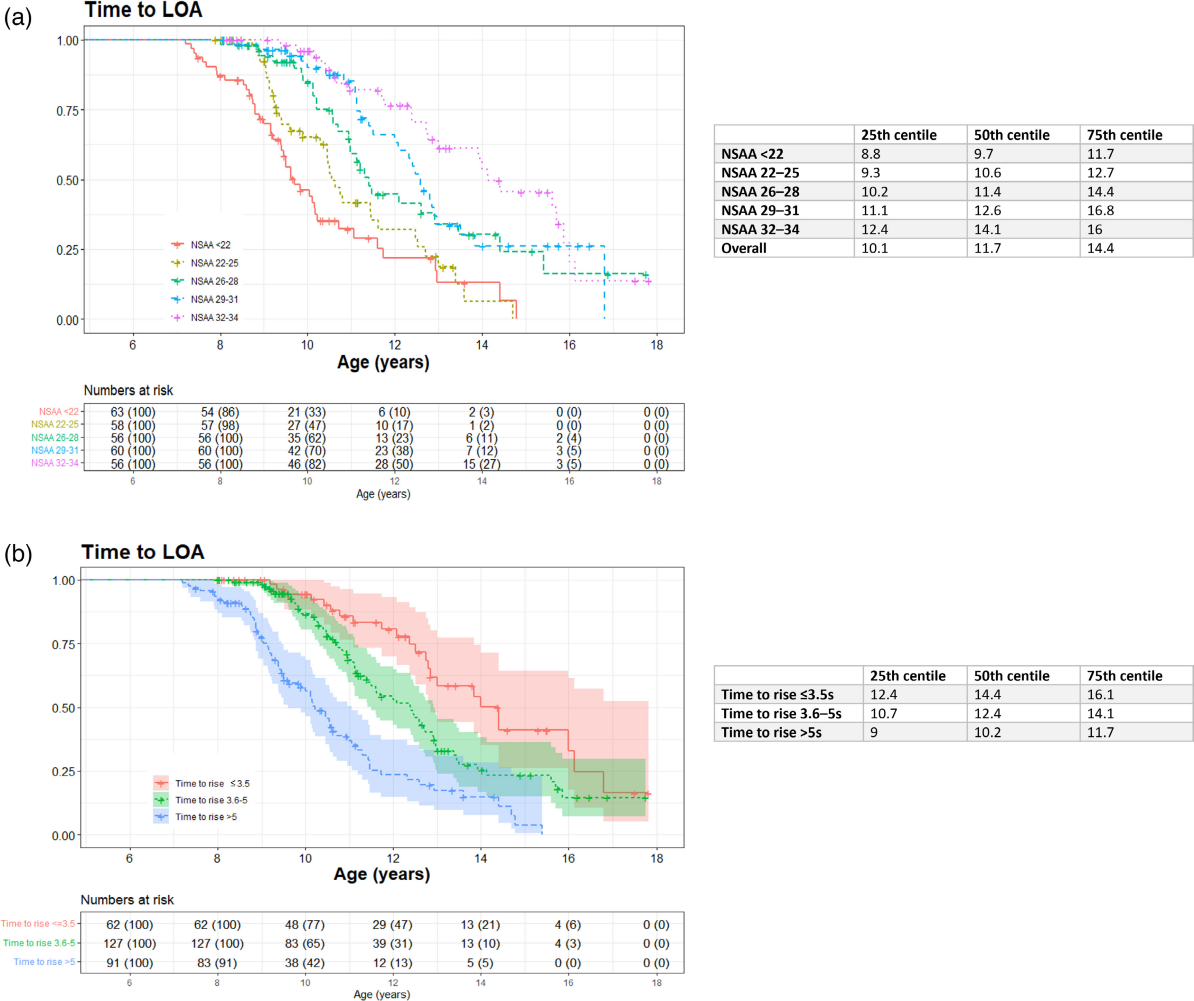
Kaplan–Meier curves showing time to loss of ambulation (LOA) according to (a) North Star Ambulatory Assessment (NSAA) group and (b) timed rise from floor (TRF)

Higher NSAA scores at baseline were associated with a later age at LOA (*p*<0.001; Fig. 2a). For patients with an NSAA score less than 22, the probability (95% CI) of being ambulant at the age of 11 years and 13 years was 0.32 (0.21–0.45) and 0.13 (0.05–0.34) respectively, while for NSAA scores of 22 to 25 the probability was 0.42 (0.29–0.6) and 0.19 (0.09–0.38). For patients with an NSAA score of 26 to 28 the probability was 0.59 (0.46–0.76) and 0.34 (0.21–0.55), while for an NSAA score of 29 to 31 the probability was 0.85 (0.75–0.96) and 0.34 (0.21–0.54), and finally for an NSAA score of 32 to 34 the probability was 0.82 (0.71–0.94) and 0.61 (0.47–0.79) respectively. None of the patients with a baseline NSAA score less than 26 were ambulant after 15 years.

TRF at baseline was positively associated with age at LOA (hazard ratio 1.13; 95% CI 1.09–1.17, *p*<0.001). When considered as TRF groups, a faster TRF at baseline was also significantly associated with an older age at LOA (*p*<0.001; Fig. 2b). In particular, the median (IQR) age at LOA for patients rising from the floor in no more than 3.5 seconds, from 3.6 to 5 seconds, and more than 5 seconds was 14 years 5 months (12 years 5 months–16 years 1 month), 12 years 5 months (10 years 8 months –14 years 1 month), and 10 years 2 months (9 years–11 years 11 months) respectively.

#### Genotype and LOA

Descriptively, no apparent differences were found except for individuals harbouring duplications and pathogenic variants amenable to exon 44 skipping, who seemed to reach LOA at a later median age than the other genotypes (Table 2 and Figure S2).

**TABLE 2:** Genotype, baseline North Star Ambulatory Assessment (NSAA) score, and age at loss of ambulation (LOA)

	Factor	*n* (%)	Median NSAA baseline (IQR)	Median TRF baseline, s (IQR)	Median age at LOA, years:months (IQR)
*DMD* genotype	Exon 44 skipping amenable deletion	22 (7)	30 (27–31) range 16–34	3.5 (2.9–4.1)	12:10 (11:1–13:10)
	Exon 45 skipping amenable deletion	26 (9)	25 (19–30) range 9–34	3.8 (3.2–5.5)	11:1 (10:0–15:7)
	Exon 51 skipping amenable deletion	41 (14)	27 (22–31) range 13–34	4.1 (3.1–5.7)	11:1 (9:4–12:7)
	Exon 53 skipping amenable deletion	24 (8)	25 (21–30) range 8–33	4.6 (3.3–5.6)	12:5 (9:11–14:10)
	Exon 55 skipping amenable deletions	14 (5)	28 (25–31) range 19–34	4.5 (2.5–6.3)	–
	Exon 50 skipping amenable deletions	10 (3)	25 (23–28) range 21–33	3.9 (3.4–4.9)	–
	Other out‐of‐frame deletion	61 (21)	27 (24–30) range 11–34	4.2 (3.5–5.6)	11:6 (10:2–13:11)
	Other out‐of‐frame duplication	27 (9)	28 (22–33) range 15–34	4.0 (3.1–4.6)	12:11 (11:7–15:11)
	Nonsense mutations	48 (16)	27 (21–31) range 10–34	4.7 (3.2–5.8)	10:8 (9:11–14:5)
	Other frameshifting or unknown mutation	30 (10)	26 (22–30) range 10–33	4.9 (3.4–5.9)	11:4 (10:1–14:8)

Note

Patients amenable to skipping of multiple exons (such as skip exons 50 and 52) were included in both groups (see Figure S1). TRF, timed rise from floor.

#### Corticosteroid regimen at baseline and LOA

The median (IQR) age at LOA of patients treated with daily versus intermittent corticosteroid regimens was 12 years 5 months (10 years 2 months–14 years 10 months) versus 11 years 5 months (10 years 1 month–13 years) respectively (*p*=0.11). The event LOA was recorded in 81 out of 141 and 41 out of 83 patients respectively (Figure S3).

### Multivariable analysis

We considered the simultaneous effects of baseline factors (NSAA, TRF, and corticosteroid regimen) on LOA to assess whether these were still independently associated with outcome or whether effects were attenuated after adjustment (Table S1). Overall, corticosteroid regimen and TRF were the most important factors (*p*=0.011 and *p*=0.007 respectively), with intermittent regimen and TRF longer than 5 seconds associated with earlier LOA. NSAA group 32 to 34 at baseline was also important (*p*=0.06), being associated with delayed LOA. After adjusting for steroids and NSAA score we found that, on average, male children with a longer TRF (>5s) had an increased hazard ratio of 1.96 in losing ambulation, meaning a 96% higher risk compared with those having a rise time between 3.6 and 5 seconds. Conversely, those with shorter baseline rise times of less than 3.5 seconds had an 18% lower risk of losing ambulation than those with rise times 3.6 to 5 seconds over a similar period.

Using the middle group (NSAA scores between 26 and 28 units) as reference, male children with a lower NSAA score comprising between 22 and 25 units had a 26% increased risk of losing ambulation. Furthermore, young males with an even lower NSAA score of less than 22 units at baseline had a 53% greater risk of losing ambulation over a similar period. For patients in the higher NSAA score categories at baseline there was a risk reduction in losing ambulation of 30% for those with scores between 29 to 31 and 48% for those with scores greater than 31, compared with those scoring NSAA 26 to 28.

Patients on daily regime at baseline had a 41% reduction in the risk of losing ambulation compared with those on intermittent regime, after adjusting for motor function outcomes (*p*=0.01).

## DISCUSSION

DMD may be considered a rather homogeneous disorder with reference to the lifespan of a typical individual. However, if we ‘change lens’ to focus on different developmental stages, an important phenotypic variability is revealed. Although a few studies have tried to identify predictive factors of motor deterioration^25^, ^26^ and to establish the risk of functional losses over a 24‐month period,^5^, ^11^, ^27^ we still have limited knowledge about what will determine individual long‐term outcomes, particularly when patients are assessed before decline of their motor abilities. Therefore, we aimed to explore whether selected factors recorded at an early stage of the disease affect age at LOA. Importantly, we intended to generate results that could be easily transferred to clinical practice.

Given the heterogeneity of the 6‐minute walk test before 7 years of age,^19^ we focused on the total NSAA total score and TRF, which are two of the most commonly used outcome measures in clinical practice.^5^ Male children with DMD achieve their highest NSAA score at a mean (SD) age of 6 years 10 months (8 months);^20^ thus we established the pragmatic window of 6 years to 7 years 6 months as baseline. At this time point, we found a clear correlation between NSAA and TRF (*r=*−0.64). We demonstrated for the first time that the best motor performance recorded in this age range (both in terms of NSAA score and TRF) is associated with age at LOA. Other studies have provided evidence that several functional measures can serve as predictors of motor deterioration; for instance, young males with a 6‐minute walk test less than 350 metres or TRF values of 5 seconds or longer are likely to experience a decline in function, and those with TRF values of at least 10 seconds are at risk of losing ambulation during the following 24 months.^11^, ^14^ Another important lesson provided by other research groups is that the addition of timed tests to conventional functional scales may improve prediction of short‐term trajectories.^28^ Nevertheless, most of these studies included older individuals who were closer to achieving major disease milestones such as LOA, with unsurprisingly higher TRF values or lower NSAA/6‐minute walk test scores. Here we suggest that, in contrast to the 6‐minute walk test, both NSAA and TRF may also be considered as early predictors of age at LOA. This observation is in keeping with previous findings showing that the lower the peak NSAA score a patient with DMD achieved (and the younger the age at which this peak was reached), the earlier their ambulatory capability started declining.^6^ Moreover, in the same study, those patients who achieved an NSAA score of no more than 5 beyond the age of 15 years had higher baseline NSAA values than individuals having earlier loss of ambulatory function. It should be noted, though, that the better‐performing group was older (mean baseline age 8 years 4 months vs overall 7 years 1 month), potentially introducing an interpretation bias, but also suggesting that preservation of motor function later into the first decade could be a strong additional prognostic factor for LOA.

When considering functional measures alone, we observed a hazard increase of 10% of losing ambulation over the same time for every second increase in the recorded baseline TRF. Those individuals with a peak raw NSAA score greater than 31 had 0.82 and an approximate probability of 0.6 of being ambulant after the age of 11 and 13 years respectively. This means that male patients with peak NSAA scores of 32, 33, and 34 may have close to double the chances of being ambulant at the age of 13 years compared with those having a peak NSAA score of 26, 27, or 28 (i.e. patients belonging to the ‘middle’ group).

Subsequently, we performed a multivariable analysis to assess whether these measures, together with corticosteroid regimen at baseline, were still independently associated with outcome. The effect of all three factors remained significant. For example, those individuals scoring lowest (<22) or highest (>31) NSAA values had an increase/decrease of approximately 50% in the risk of losing ambulation compared with the intermediate group (26–28), regardless of baseline TRF and corticosteroid regimen. These data allow us to provide a rational and quantitative framework to the general notion that patients with milder symptoms are likely to walk for longer. However, they also suggest that a non‐negligible proportion of patients (~40%) may lose ambulation before the age of 13 years despite presenting with very good baseline motor abilities, adding to the difficulty of giving a prognosis to younger patients. This seemingly contrasting result could be explained by the complexity of the pathogenesis underlying DMD, with different mechanisms each associated with potential for modifiers (including muscle damage, inflammation, and regeneration failure), and theoretically playing different roles in specific stages of the disease.^29^

This work is a first step in assessing potential early clinical predictors for LOA. Although TRF seemed more informative than NSAA in multivariable analysis, the combination of the two to form an inclusive predictive model was beyond the scope of this study. Moreover, the caveat is that in such a rare (and heterogeneous) disorder it is extremely difficult to create a reliable predictive model.

Several other factors could contribute to modify the natural history of DMD, particularly corticosteroid treatment and genotype. While solid evidence has been provided about the beneficial effects of corticosteroid‐treatment (vs naive),^8^ we have limited knowledge about the differences given by different corticosteroid types/regimens owing to potential biases introduced in real‐world practice. As an example, the earlier initiation of steroids has been variably associated with a higher rate of improvement at younger than 7 years of age,^5^ but also with worse outcomes, probably reflecting the real‐life scenario in which patients with more severe forms of the disorder are offered steroids earlier than later presenters.^6^ We confirmed here that the use of daily versus intermittent regimen at baseline was associated with a lower risk of LOA, although this advantage had a smaller magnitude than previously reported.^7^ Interestingly, we also observed that a good proportion of patients were on a corticosteroid dose that was more than 20% less than the optimal dose pro‐kilo indicated for deflazacort and prednisolone (around 30%). The clinical significance of this is unclear^30^ but researchers should consider this pitfall which is rarely depicted in other observational studies.^18^, ^31^

Lastly, while we added to the evidence that patients harbouring a pathogenic variant amenable to the skipping of exon 44 show a milder phenotype,^5^, ^9^, ^13^ we did not find solid evidence linking variants amenable to other exon skipping to worse/better outcomes (Fig. S2b). This is in keeping with existing conflicting data,^6^, ^9^, ^13^, ^32^ confirming that, with the exception of a few genotypes in which residual dystrophin expression can be observed, dystrophin mutations are not the primary driver of disease progression in patients with DMD.

This study has several limitations. First, the NSAA database describes real‐world practice well but has some missing data. Given the inability to precisely define the NSAA peak (approximately half of our patients lacked an NSAA score <6 years), we needed to define a pragmatic age boundary as baseline. By considering the highest score in a time window shorter than 18 months, we tried to minimize potential biases introduced by poor child compliance at the time of the assessment. In multivariable analysis, we considered corticosteroid regimen at baseline, but close to 40% of patients later switched steroid type and/or regimen. Lastly, all patients included in the study were subject to the standards of care of a single country (UK). While on one hand we expect a significant ethnic heterogenicity, this cohort might not be representative of other DMD populations.

In conclusion, both NSAA and TRF recorded at the age of expected peak are significant determinants of age at LOA in male children with DMD, the latter being more important even when recorded early in the disease course. This work highlights the multiple variables that can affect the design of clinical trials and the importance of including baseline measures in future retrospective studies whose main endpoint is LOA. Finally, we provide useful information that will help clinicians to counsel families in a clinical setting, particularly when discussing prognosis.

## Supporting information


**Figure S1:** Patients’ selection and DMD variants.

**Figure S2:** Rainbow plot representing the distribution of NSAA scores at baseline according to genotype and Kaplan–Meier curve showing time to LOA according to genotype.

**Figure S3:** Kaplan–Meier curve showing time to LOA according to corticosteroid regimen.

**Table S1:** Cox regression parameters for the time‐to‐event analysis of LoA.

## Data Availability

The data that support the findings of this study are available from the corresponding authors, upon request.
