# Supplementary material for: Peak functional ability and age at loss of ambulation in Duchenne muscular dystrophy
Source: Dev Med Child Neurol. 2022 Feb 14;64(8):979–88. doi: 10.1111/dmcn.15176 (PMC9303180; doi:10.1111/dmcn.15176)
Supplement: Supplementary file 1 — Figure S1: Patients’ selection and DMD variants. [file DMCN-64-979-s003.docx]

**Supplementary Figure 1.** **Patients' selection and DMD variants**


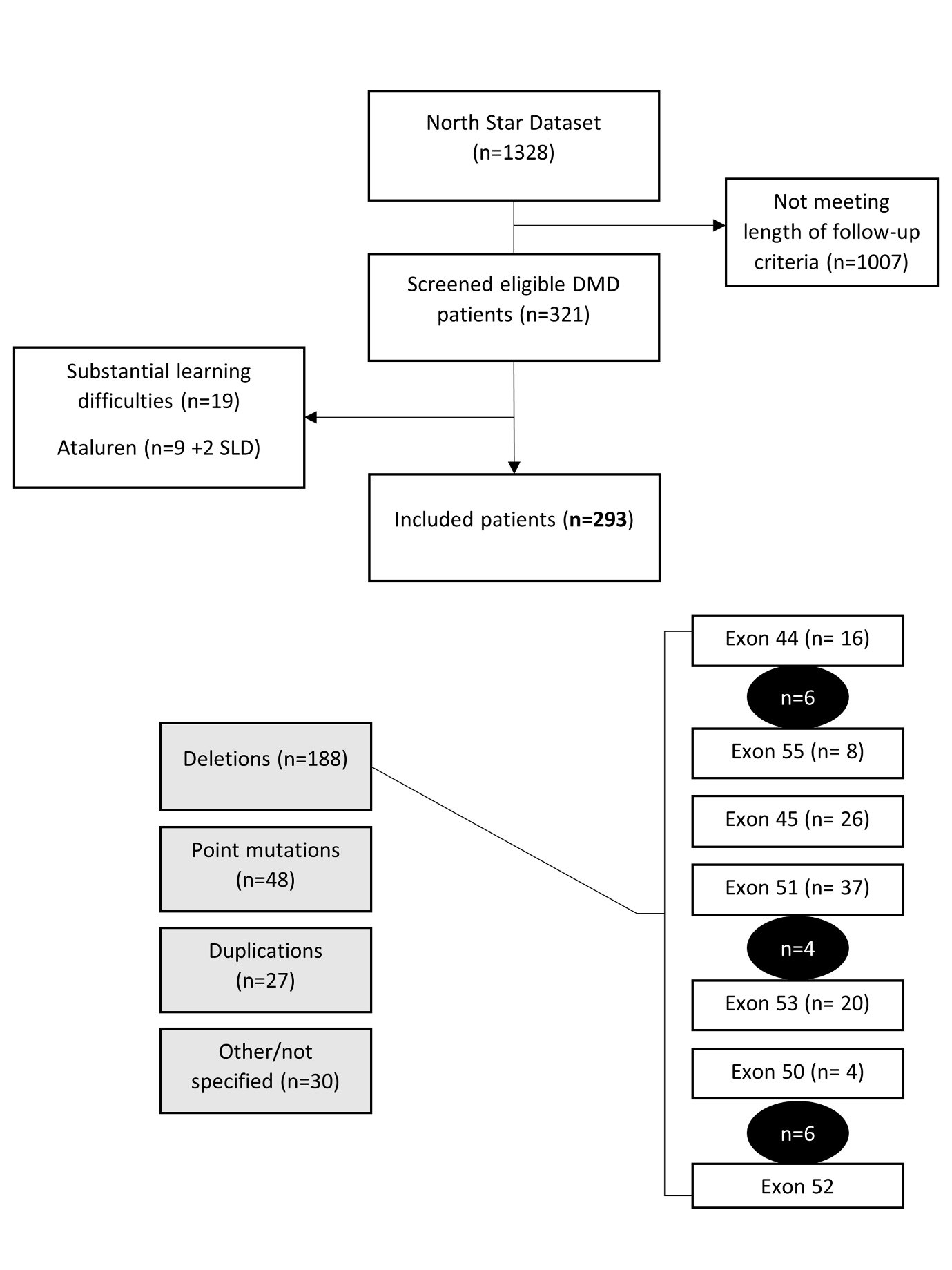
Top diagram displays the patients’ selection process. The lower diagram displays the number of patients according to type of mutation (left) and number of patients amenable to specific exon skipping (right). Numbers in black ovals: patients amenable to different types of skipping (e.g., exon 51 🡪 37 + 4= total 41). Sixty-four percent of patients had large deletions of the DMD gene, 16% had point mutations, 9% large duplications and 10% either small frameshift rearrangements or unknown pathogenic variants. Only 3 patients had duplication of exon 2, one duplication of exon 3-7 and none had exon 3-7 deletion, a DMD variant that has been associated with milder phenotypes^26^. A total of 41 patients (14%) had variants amenable to exon 51 skipping. Four of these had deletions also amenable to exon 53 skipping. The second most frequent group of skippable variants was exon 45 skipping (9%), followed by exon 53 (8%), exon 44 (7%), exon 55 (5%), exon 50 (3%) and exon 52 (2%).
