# Supplementary material for: Peak functional ability and age at loss of ambulation in Duchenne muscular dystrophy
Source: Dev Med Child Neurol. 2022 Feb 14;64(8):979–88. doi: 10.1111/dmcn.15176 (PMC9303180; doi:10.1111/dmcn.15176)
Supplement: Supplementary file 2 — Figure S2: Rainbow plot representing the distribution of NSAA scores at baseline according to genotype and Kaplan–Meier curve showing time to LOA according to genotype. [file DMCN-64-979-s001.docx]

**Supplementary Figure 2.** Rainbow plot representing the distribution of NSAA scores at baseline according to genotype (A). Kaplan-Meier curve showing time to LOA according to genotype.
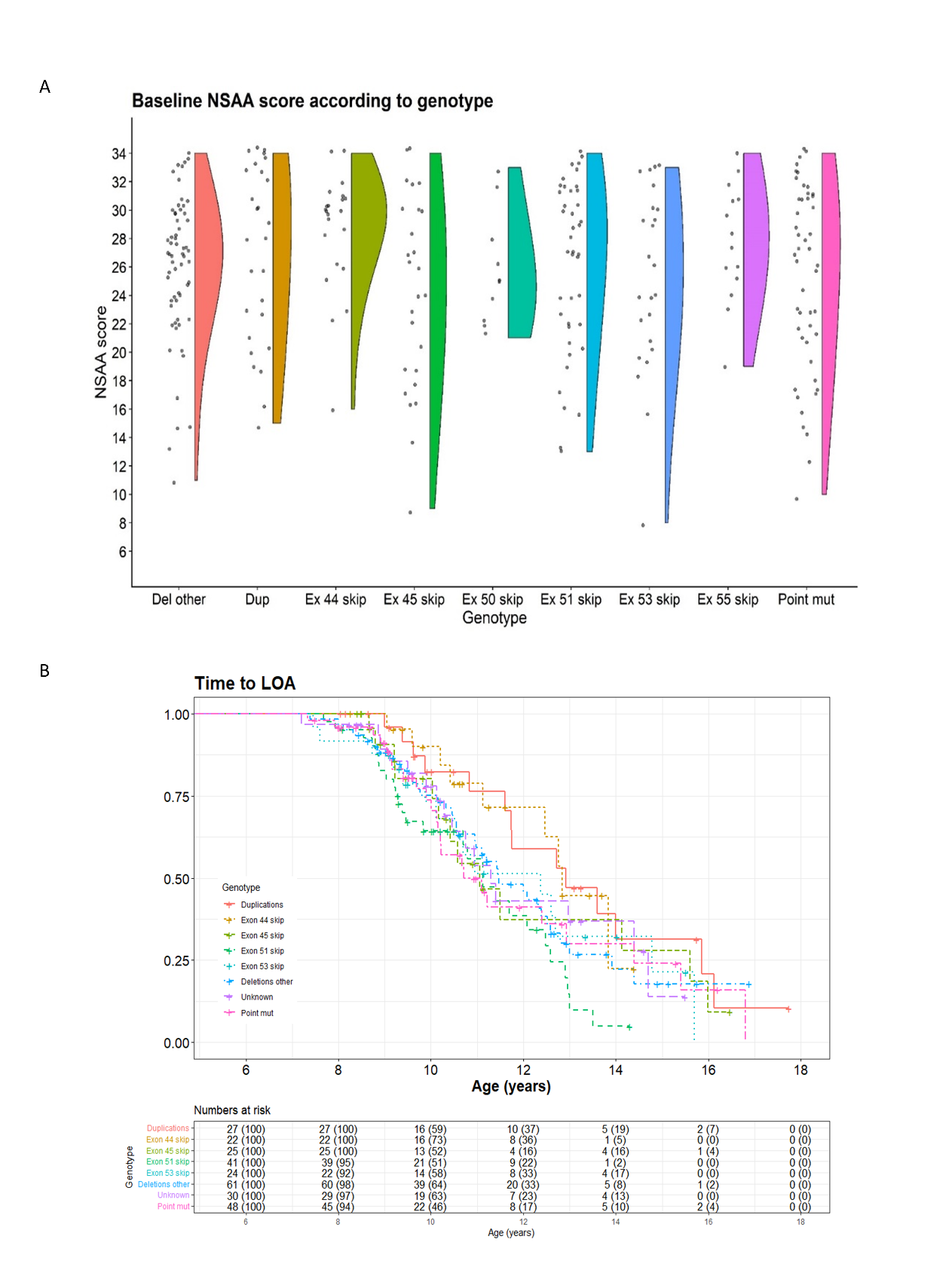
*Abbreviations: Del, deletion; Dup, duplication; exon “X” skip, variant amenable to exon “X” skipping, LOA, loss of ambulation; NSAA, North Star Ambulatory Assessment; Point mut; point mutation; TRF, Timed Rise from Floor.*
