# Supplementary material for: Peak functional ability and age at loss of ambulation in Duchenne muscular dystrophy
Source: Dev Med Child Neurol. 2022 Feb 14;64(8):979–88. doi: 10.1111/dmcn.15176 (PMC9303180; doi:10.1111/dmcn.15176)

**Supplementary Figure 3.** Kaplan-Meier curve showing time to LOA according to corticosteroid (CS) regimen. Daily vs intermittent regimen was defined by the regimen used for >50% of time before LOA, regardless CS type.


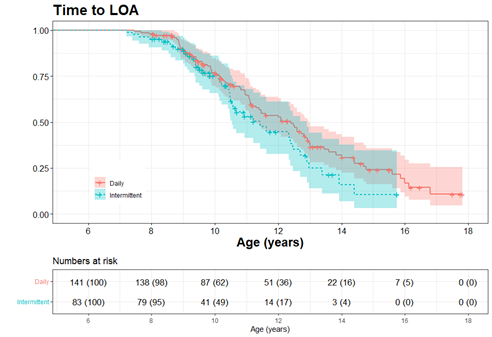

Supplement: Supplementary file 3 — Figure S3: Kaplan–Meier curve showing time to LOA according to corticosteroid regimen. [file DMCN-64-979-s004.docx]
