# Supplementary material for: Peak functional ability and age at loss of ambulation in Duchenne muscular dystrophy
Source: Dev Med Child Neurol. 2022 Feb 14;64(8):979–88. doi: 10.1111/dmcn.15176 (PMC9303180; doi:10.1111/dmcn.15176)
Supplement: Supplementary file 4 — Table S1: Cox regression parameters for the time‐to‐event analysis of LoA. [file DMCN-64-979-s002.docx]

**SUPPLEMENTARY MATERIAL**

**Supplementary Table 1. Cox regression parameters for the time-to-event analysis of LoA**

| Variable | Hazard Ratio | 95% CI | P -value |
| --- | --- | --- | --- |
| Baseline CS regimen  *Intermittent*  *Daily* | *1*  *.5858554* | *.3878032 .8850534* | ***0.011*** |
| Baseline NSAA group  *NSAA <22*  *NSAA 22-25*  *NSAA 26-28*  *NSAA 29-31*  *NSAA 32-34* | *1.530288*  *1.255326*  *1*  *.7006613*  *.5207411* | *.8148407 2.873913*  *.7086082 2.223856*  *.3759582 1.3058*  *.2615223 1.036896* | *0.186*  *0.436*  *0.263*  ***0.063*** |
| Timed rise group  *<3.5 s*  *3.6-5 s*  *>5 s* | *.8206209*  *1*  *1.957949* | *.4541442 1.48283*  *1.201986 3.189359* | *0.513*  ***0.007*** |
| Age at CS start | *1.042381* | *.809847 1.341683* | *0.747* |

*Table legend. The proportional hazards assumption is satisfied for the above model. Overall test for baseline NSAA group, P = 0.05. Overall test for Timed rise group, P = 0.01*
